# Postoperative Radiotherapy and the Role of Regional Lymph Node Irradiation in Localized Merkel Cell Carcinoma: A Single-Center Retrospective Analysis

**DOI:** 10.3390/cancers14246140

**Published:** 2022-12-13

**Authors:** Lisa-Antonia Dinges, Tanja Eichkorn, Sebastian Regnery, Juliane Hörner-Rieber, Jürgen Debus, Jessica C. Hassel, Kristin Lang

**Affiliations:** 1 Department of Radiation Oncology, Heidelberg University Hospital, Im Neuenheimer Feld 400, 69120 Heidelberg, Germany; 2 Heidelberg Institute for Radiation Oncology (HIRO), Heidelberg University, Im Neuenheimer Feld 400, 69120 Heidelberg, Germany; 3 National Cancer Center for Tumor Diseases (NCT), 69120 Heidelberg, Germany; 4 Clinical Cooperation Unit Radiation Oncology, German Cancer Center (DKFZ), 69120 Heidelberg, Germany; 5 Department of Dermatology, University Hospital of Heidelberg, 69120 Heidelberg, Germany

**Keywords:** Merkel cell carcinoma, adjuvant radiation therapy, postoperative radiation therapy, elective lymph node irradiation

## Abstract

**Simple Summary:**

Merkel cell carcinoma (MCC) is a rare, malignant primary skin tumor with high rates of recurrence and stage adapted 5-year overall survival rates around 66–75% at stage I, 50–60% at stage II, 42–52% at stage III and 17–18% at stage IV. Wide local tumor excision is indicated for localized disease, usually followed by postoperative radiotherapy of the tumor bed. A positive sentinel lymph node biopsy (SLNB) is considered a poor prognostic factor, but the only randomized controlled trial on elective lymph node irradiation was in times before introduction of SLNB in MCC, hence the management of the regional lymph nodes with respect to SLNB is currently not clear. The aim of this study was to analyze the pattern of relapse of patients with MCC that underwent surgery and postoperative radiotherapy at our institution and to determine the role of elective radiotherapy to regional lymph nodes with respect to SLNB results.

**Abstract:**

The aim of this study was to analyze the pattern of relapse of patients with Merkel cell carcinoma (MCC) that underwent resection of the primary tumor site and postoperative radiotherapy at the Department of Radiation Oncology of Heidelberg University and to determine the role of the elective radiotherapy of regional lymph nodes with respect to SLNB results. A total of 57 patients were included in the present retrospective analysis. A total of 33 patients had additional lymph node irradiation (LNI); 24 had postoperative radiotherapy of the tumor bed only. Median follow-up was 43 months. Recurrence rate of the total cohort was 22.8%. Most relapses (69%) occurred in the regional nodes. Cumulative infield-tumor recurrence rate was low with 5.3%. Regional recurrence was more frequent in the cohort without LNI with 85.7% versus 37.5% with LNI. These results were similar for patients with negative sentinel lymph node (SLN) only with 80% regional relapses for those without LNI versus 33% with LNI. In conclusion, our data show that regional recurrence is the most frequent site of relapse in stage I-III MCC treated with curative intended postoperative radiotherapy and that elective irradiation of the regional lymph nodes reduces the risk of regional relapse even if the SLN was negative.

## 1. Introduction

Merkel cell carcinoma (MCC) is a rare, malignant primary skin tumor with epithelial and neuroendocrine differentiation, which typically occurs at an older age with a median age at diagnosis of approximately 70 years [1]. The localization is mostly at sun-exposed areas, particularly the head and neck area, and, less frequently, the extremities [2]. According to the most recent staging system of the American Joint Committee on Cancer (AJCC) four clinical stages are differentiated: stage I and stage II include localized disease without nodal spread and a primary lesion of ≤2 cm or >2 cm; stage III is localized disease with regional nodal spread and stage IV includes metastatic disease beyond local lymph nodes [1]. Previous studies show 5-year survival rates around 66–75% at stage I, 50–60% at stage II, 42–52% at stage III and 17–18% at stage IV [2,3,4] with locoregional recurrence or distant metastasis mostly occurring within the first two years of initial diagnosis [5,6]. Due to its aggressiveness, multimodal tumor therapy of MCC is indicated, even at an early stage. Wide local tumor excision with safety margins of 1–2 cm is indicated for localized disease [7,8,9,10], usually followed by postoperative radiotherapy of the tumor bed [11,12,13,14,15,16,17]. However, the management of the regional lymph nodes remains less clear. To our knowledge there is only one randomized controlled trial evaluating efficacy and safety of a prophylactic adjuvant radiotherapy on the regional nodes [16]. Jouary et al. compared the prophylactic adjuvant radiotherapy of the regional nodes with observation only at stage I MCC treated by wide local excision and irradiation of the tumor bed. They found a significantly reduced probability of regional recurrence (PRR) with 17% in the observation arm vs. 0% in the treatment arm and good tolerability of the treatment. But the improved PRR did not transfer to significant improvements in overall survival and progression-free survival, which however might be due to premature trial end (inclusion of only 83 of the 210 planned patients) because of a drop in recruitment due to the introduction of the sentinel lymph node biopsy (SLNB) in the early 2000s, which was an exclusion criterion for trial participation [16]. The study of Jouary et al. therefore only provides data for patients with clinical negative lymph node staging. Without any scientific evidence to date the NCCN guidelines recommend for patients with a positive SLNB surgical lymphadenectomy (LAD) and/or radiation therapy to the nodal basin. For SLNB negative patients the nodal basin should be observed and radiotherapy considered in high-risk patients only. However regional recurrence is observed in 10–25% of patients with negative SLNB, which might either be explained by a relevant false negative rate of SLNB or the general aggressiveness of MCC. This is in line with some small retrospective studies, that suggest that elective radiation therapy of the regional nodes might even be advantageous in case of negative SLNB [5,18,19].

The aim of this study was to analyze the general outcome and pattern of relapse of patients with MCC that underwent surgery and postoperative radiotherapy at our institution and to determine the role of elective radiotherapy to regional lymph nodes with respect to SLNB results.

## 2. Patients and Methods

### 2.1. Patients and Treatment Characteristics

For this retrospective analysis we reviewed all patients with MCC, that underwent radiotherapy at Department of Radiation Oncology of the Heidelberg University Hospital between January 2010 and December 2020. Included were patients with locoregional disease (AJCC-stage I-III), that had resection of the primary tumor +/- SLNB or LAD, followed by either postoperative radiotherapy of the tumor bed only or combined with elective radiotherapy of the regional lymph nodes. We identified 57 patients. 46 of them were staged surgically (37 patients had SLNB, 5 patients had SLNB followed by LAD, 4 patients had LAD). A total of 33 of them underwent radiotherapy to the primary site plus additional radiotherapy to the regional lymph node basin, and 24 patients had postoperative radiotherapy of the tumor bed only.

Initial excision biopsy of the MCC was often performed at an external institution, resulting in 19 cases, in which the exact size of primary tumor remains unclear due to an imprecise external pathologic report (listed as AJCC-stage ≤ II). The institutional treatment policy after excision biopsy was re-excision with margins of 1–2 cm if functionally possible. The radiotherapeutic treatment was either external beam radiotherapy to a total dose of 50–60 Gy in 25–30 fractions with single doses of 1.8–2.1 Gy or brachytherapy to a total dose of 36–48 Gy in 9–13 fractions with single doses of 3.5–4.0 Gy. Brachytherapy was only used for treatment of the tumor bed, treatment of regional lymph nodes was always applied via external beam radiotherapy (EBRT). 

### 2.2. Objectives

Primary endpoints were overall survival (OS) and progression-free survival (PFS). Secondary endpoints were pattern of relapse of the regional nodes as well as local, regional and distant recurrence-free survival (RFS). 

Local, regional and distant RFS were defined as the time from initial diagnosis to the first (local, regional or distant) date of recurrence or date of last follow-up. Recurrence at other sites and death were censored. PFS was defined as the time from initial diagnosis to the patient’s first recurrence or date of last follow-up. Any recurrence of MCC and death of any cause were considered an event. OS was defined as the time between the date of initial diagnosis and date of last follow-up. Death of any cause was considered an event.

Of special interest was the comparison of patients, who received irradiation of the tumor bed only versus patients who underwent additional elective irradiation of the regional lymph nodes. This comparison was conducted for the total cohort as well as for the subgroup with negative SLNB only.

### 2.3. Statistical Analysis

Descriptive statistics for baseline variables and for endpoints include median and range for continuous variables and absolute and relative frequencies for categorical variables. For normally distributed variables, a paired sample t-test was used to test for statistical differences between data sets, otherwise the Wilcoxon signed rank test or the exact Fisher’s test were applied. Univariate survival analysis of categorical variables was conducted with the Kaplan-Meier method together with the Log-Rank test for statistical significance. Univariate and multivariate Cox proportional hazards regression model was conducted to assess the association of baseline variables on survival outcome. For multivariate analysis all tested patient, tumor and treatment factors were chosen based on clinical relevance. A *p*-value less than 0.05 was considered statistically significant. R Version 4.0.3 was used to conduct all statistical analyses. 

### 2.4. Ethics

All analyses were performed following institutional guidelines and the Declaration of Helsinki of 1975 in its most recent version. Ethics approval for the study was granted by the Heidelberg University ethics committee on August 2nd, 2021 (#S-587/2021). Patient confidentiality was maintained by anonymizing patient data to remove any identifying information.

## 3. Results

### 3.1. Patient Characteristics 

Median age of the cohort was 73 years (range 50–89 years). A total of 40 patients (70.2%) had stage I or II MCC. Only 17 patients (29.8%) had a locally advanced stage III MCC. A total of 19 patients (33.3%) had the primary tumor localization in the craniofacial area; 6 patients (10.5%) at the body trunk and 32 patients at the extremities (56.2%). Of the 57 patients, 12 (21.1%) had a positive sentinel lymph node biopsy (SLNB) without macroscopic lymph node involvement and 30 (52.6%) had a negative result of the SLNB. A total of 15 patients (26.3%) did not receive SLNB before the postoperative radiotherapy. Lymph node dissection was rare with only 9 cases (15.8%) and the great majority of the patients (91.2%) had a R0 resection status (Table 1). 

The majority of patients (78.9%) received an external beam radiotherapy (EBRT) with a total dose of 50–60 Gy in 1.8–2.1 Gy single doses. Twelve patients (21.1%) received postoperative brachytherapy of the tumor bed only. Thirty-three patients (57.9%) received a lymph node irradiation (LNI) of the regional lymph node basin. This was done in all SLNB positive cases and in 10/30 (33.3%) of patients with negative SLNB (Table 1).

### 3.2. Oncologic Outcome and Patterns of Relapse of the Total Cohort

**Figure 1:**
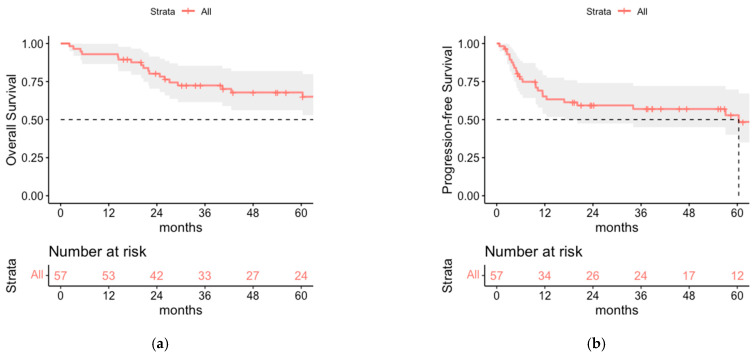
(**a**) Overall survival (OS) and (**b**) progression-free survival (PFS) of the total cohort (n = 57). Median OS was 112 months, median PFS was 60.4 months.

**Figure 2:**
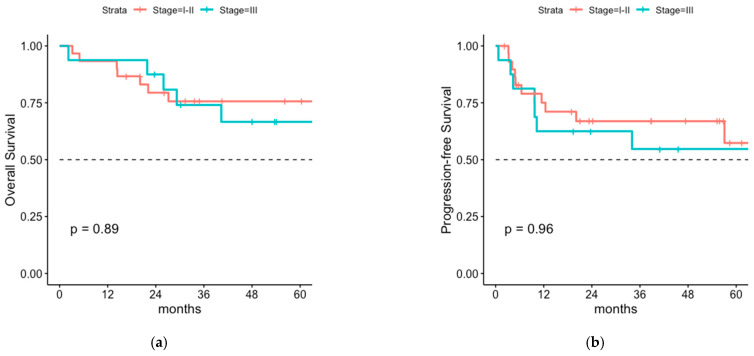
(**a**) Stage-adapted overall survival and (**b**) stage-adapted progression-free survival of surgically staged patients (n = 46).

**Figure A1:**
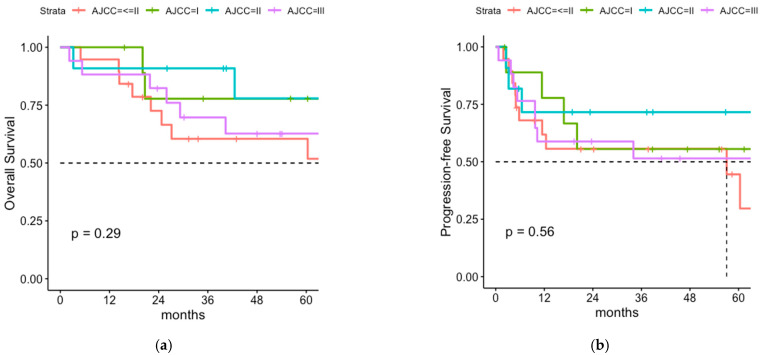
Stage adapted (**a**) OS and (**b**) PFS of the total cohort for stage I, stage II, stage ≤ II and stage II, according to AJCC-staging classification (n = 57).

**Figure A2:**
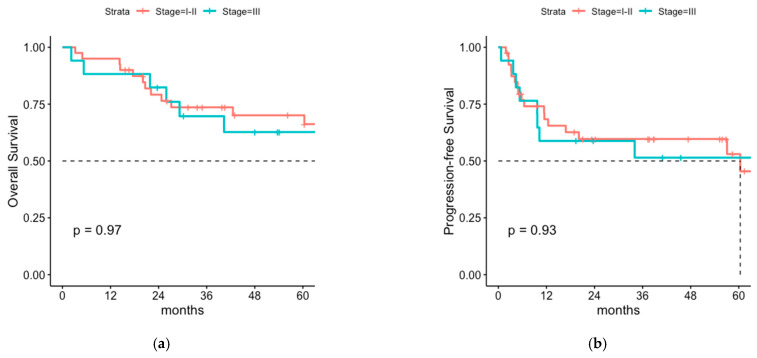
(**a**) Stage-adapted OS and (**b**) PFS of the total cohort for stage I-II and stage III, according to AJCC-staging classification (n = 57).

**Figure A3:**
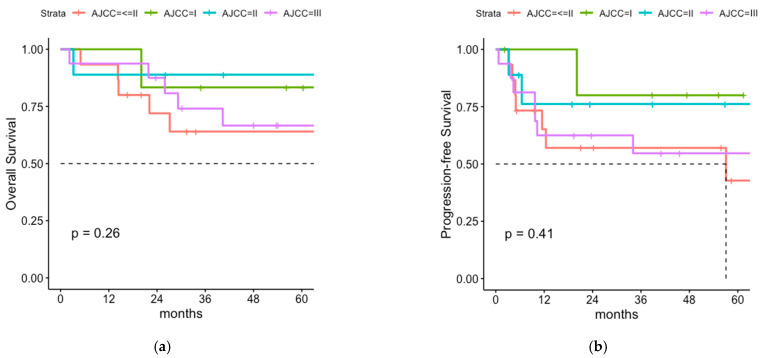
Stage-adapted (**a**) OS and (**b**) PFS of the subgroup of surgically staged patients only for stage I, stage II, stage ≤ II and stage III, according to AJCC-staging classification (n = 46).

After a median follow-up time of 43 months (range 2–134) 1-, 3- and 5-year OS rate was 93.0% (95% CI 86.6–99.9%), 72.5% (95% CI 61.5–85.4%) and 67.8% (95% CI 65.1–81.9%) respectively (Figure 1). The median OS was 9.3 years. PFS rates were 65.2% (95% CI 53.7–79.2%) after 1 year and 57.0% (95% CI 45.0–72.1%) and 52.9% (95% CI 40.1–69.8%) after 3 and 5 years, respectively (Figure 1). Median PFS was 5 years. Interestingly, no differences in PFS and OS were seen for stage I-II MCC versus stage III MCC, even for the subgroup of surgically staged patients only (Figure 2, Figure A1, Figure A2 and Figure A3).

A total of 13 of the 57 patients (22.8%) experienced tumor recurrence within the median follow-up period of 43 months. The most frequent site of relapse was the regional lymph node basin in 9/13 (69%) of the patients, although one of them had additional local recurrence and two of them additional distant recurrence. Cumulative infield-tumor recurrence rate was low with 5.3% (3/57 patients) of the total cohort.

### 3.3. Comparison of Postoperative Radiotherapy with or without Regional Lymph Node Irradiation (LNI) 

To analyze the efficacy of radiotherapy to the regional lymph node basin we compared patients who received radiotherapy of the tumor bed only with the ones that received an additional regional lymph node irradiation (LNI). Due to the retrospective design of this study, patient characteristics were significantly different in both cohorts in regard to the presence of a positive sentinel lymph node and consecutively in regard to the AJCC-stage with 51.5% patients in the cohort with LNI being staged III according to AJCC versus 0% in the cohort without LNI (*p* < 0.001). Brachytherapy was only applied in the cohort without LNI. No significant differences were seen for age, sex, resection status, primary tumor localization and whether a lymphadenectomy was performed or a sentinel lymph node biopsy was done (Table 1).

**Figure 3:**
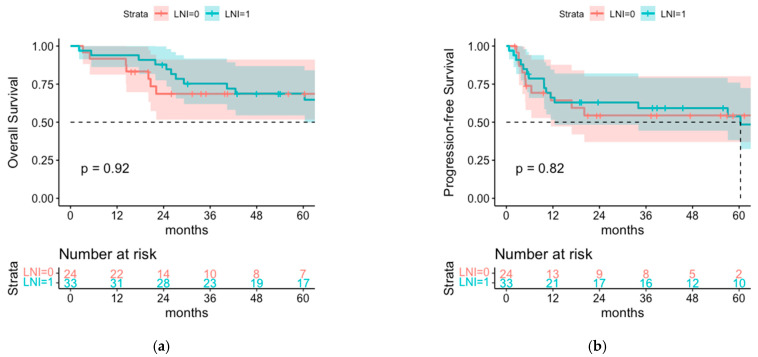
(**a**) OS and (**b**) PFS of patients who received postoperative radiotherapy of the tumor bed with additional lymph node irradiation (LNI = 1) or without lymph node irradiation (LNI = 0).

Despite the higher AJCC stage in the cohort with LNI there was no difference in OS between both groups with 1- and 3-year OS rates of 93.9% and 68.8% in the cohort with LNI and 91.7% and 68.6% in the cohort without LNI (*p* = 0.92) (Figure 3). There is also no significant difference between both groups regarding PFS with 1- and 3-year PFS of 66.1% and 59.2% versus 64.3% and 54.4% in the group with and without LNI, respectively (*p* = 0.8). 

Local recurrence (LR) was genuinely rare and occurred in one patient of each cohort (LR rate 3.0% in the LNI cohort versus 4.2% in the cohort without LNI). At the end of follow-up, the cumulative regional recurrence rate was 9.1% in the cohort with LNI (3/33 patients) and 25% in the cohort without LNI (6/24 patients). Distant recurrence was significantly more frequent observed in the LNI cohort (with higher AJCC stage) with a cumulative distant recurrence rate of 15.2% (5/33 patients) versus 0% in the cohort without LNI (0/24 patients, *p* = 0.049) (Table 2). This results in 85.7% of relapses being localized in the regional nodes (6/7) in the cohort without LNI versus 37.5% (3/8) of relapses being localized in the regional nodes in the cohort with LNI (*p* = 0.11) and a relative risk of 2.28 for relapse located in the regional nodes for the cohort without LNI.

**Figure 4:**
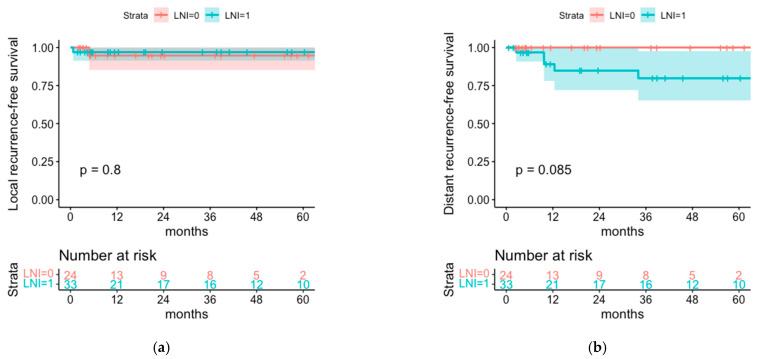
(**a**) Local and (**b**) distant recurrence-free survival of the cohorts with regional lymph node irradiation (LNI = 1) or without regional lymph node irradiation (LNI = 0).

No statistically significant differences were seen for the corresponding rates of local recurrence-free survival with 97.0% and 94.7% (*p* = 0.8) and the rates of distant recurrence-free survival with 79.8% and 100% (*p* = 0.09) for the cohort without LNI and with LNI after 5 years, respectively (Figure 4).

**Figure 5:**
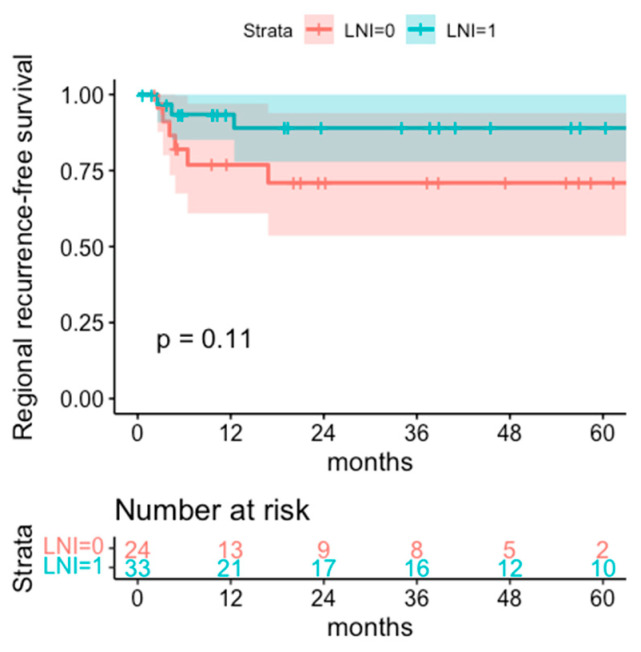
Regional recurrence-free survival of the cohorts with regional lymph node irradiation (LNI = 1) or without regional lymph node irradiation (LNI = 0).

Regional recurrence-free survival is higher in the LNI cohort with 93.4% and 89.0% (95% CI 85.0–100% and 77.8–100%) after 1 year and 3 years, respectively, versus 76.9% and 71.0% (95% CI 60.9–97.0% and 53.6–93.9%) in the cohort without LNI, however statistical significance is not reached (*p* = 0.11) (Figure 5). All tumor recurrences were observed within the first 3 years after treatment.

### 3.4. Cox Proportional Hazard Model for OS, PFS and Regional Recurrence-Free Survival

A univariate cox proportional hazard model showed a significant association of OS with the variables female sex (HR 0.31, 95% CI 0.11–0.85, *p* = 0.022) and having had SLNB done (HR 0.417, 95% CI 0.177–0.982, *p* = 0.045). A positive SLNB result was however not significantly associated with a poor prognosis (HR 1.3, 95% CI 0.39–4.31, *p* = 0.66). LNI, AJCC-stage, primary tumor localization and having had a lymphadenectomy were not significantly associated with OS. Higher age at diagnosis was the only statistically significant poor prognostic factor (HR 1.15, 95% CI 1.08–1.23, *p* < 0.001) for OS. The statistically significant results only held for age at diagnosis and female sex in the multivariate analysis (considering the following variables: LNI, having had SLNB, sex, age at diagnosis, AJCC-stage). 

A univariate cox proportional hazard model of PFS showed only a statistically significant impact of the variable age at diagnosis (HR 1.10, 95% CI 1.05–1.16, *p* = 0.0003). Univariate analysis of regional recurrence-free survival showed a positive association with lymph node irradiation, however statistical significance was not reached (HR 0.34, 95% CI 0.09–1.36, *p* = 0.13). 

### 3.5. Postoperative Radiotherapy with or without Regional LNI for Patients with Negative SLNB 

**Figure 6:**
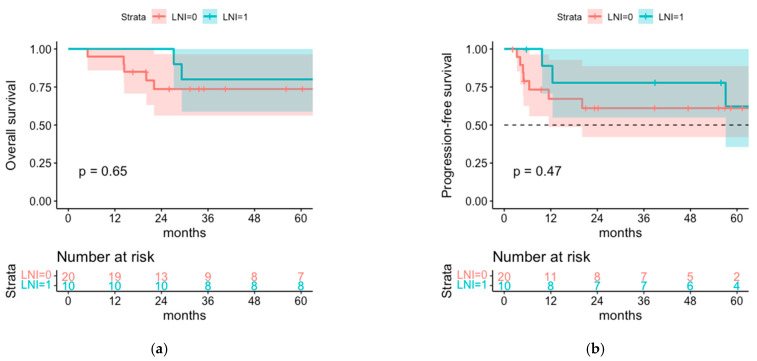
(**a**) OS and (**b**) PFS for SLNB negative patients with LNI (LNI = 1) and without LNI (LNI = 0).

Of the 30 patients that had a negative SLNB, 20 patients underwent postoperative radiotherapy of the tumor bed only and 10 received an additional radiotherapy of the regional lymph node basin. The 1- and 5-year OS rates were numerically better in the LNI cohort with 100% and 80% (95% CI 58.7–100%) versus 95% (95% CI 85.9–100%) and 73.7% (95% CI 56.2–96.6%) in the cohort without LNI. Similarly, the 1- and 3-year PFS rates were better in the LNI cohort with 88.9% (95% CI 70.6–100%) and 77.8% (95% CI 54.9–100%) versus 67.2% (95% CI 48.7–92.8%) and 61.1% (42.1–88.7%) in the cohort without LNI, but again are not statistically significant (Figure 6).

In the cohort with negative SLNB, cumulatively, six patients (20% in each cohort) had tumor progression after a median follow-up of 62.7 months (range 5–134). Relapse in the regional nodes occurred in 4/20 patients (20%) in the cohort without LNI versus 1/10 (10%) in the cohort with LNI (*p* = 0.64), resulting in 80% of relapses (4/5 sites of relapse) in the cohort without LNI being localized in the regional nodes versus 33% (1/3 sites of relapse) in the cohort with LNI (*p* = 0.33), and a relative risk of 2.4 for relapse located in the regional nodes for the cohort without LNI (Table 3).

### 3.6. Lymph Node Irradiation of Sentinel Node Positive Patients without Previous LAD

Out of the 12 patients that had a positive SLNB, only 5 patients underwent lymphadenectomy. The remaining seven patients underwent radiation monotherapy of the regional lymph nodes due to refusal of the surgery. One of these patients unfortunately experienced rapid outfield locoregional tumor recurrence of the contralateral face half under radiotherapeutic treatment. The patient was not capable for systemic treatment due to comorbidity and died within 3 months due to sudden cardiac death. Apart from this case, all other patients (6/7) that received radiation monotherapy for the microscopic lymph node involvement were recurrence-free until end of follow-up period.

## 4. Discussion

MCC is known as a rare but aggressive disease with high rates of recurrence [20,21]. While several studies provide evidence that postoperative radiotherapy of the tumor bed significantly reduces risk of recurrence [13,14,16] and is allegedly associated with better overall survival [12,17,22], the management of the regional lymph nodes remains controversial and is not well defined yet.

With this study, we aimed to provide further information on outcome and patterns of relapse for postoperative radiotherapy with or without radiotherapy of the regional lymph nodes with respect to previously performed SLNB.

Unfortunately, due to the retrospective design of the study, the exact re-classification according to AJCC-staging system was not possible in all cases. The external pathology reports often did not contain the exact tumor size, which resulted in 19 cases in which the primary tumor size was in synopsis with the re-resection pathology report <5 cm, but it remained unclear whether it was ≤2 cm or >2 cm. To differentiate these cases from the definite stage I and stage II cases, we subsumed them as stage ≤ II. Because of small patient numbers and inconclusive results in the individual stage-adapted OS and PFS (Figure 2, Figure A2), we summarized all stage I-II patients versus stage III patients in the further stage-adapted analyses.

### 4.1. Overall Survival and Progression-Free Survival of Patients with or without LNI

The 5-year OS rate of our cohort was 67% and the 5-year PFS rate 52.9%, which is in line with previous studies [2,3,4,21]. While the cumulative rate of relapse is 22.8%, the 1-year PFS is relatively low with only 65.2%. This is most likely due to the higher age of the cohort (median age 73 years) and a consequently higher number of deaths due to other causes. Interestingly the stage-adapted OS and PFS are not different for stage I-II and stage III MCC in the present study. Because of a possible bias by understaging of the patients without SLNB, we analyzed this also for the subgroup of surgically staged patients only, for which the results held. This is in contrast to several studies providing strong evidence for AJCC stage to be one of the most relevant prognostic factors for MCC [2,3,4,23] and is, in our opinion, primarily due to the different treatment approaches. All of the MCC stage III patients (100%) received postoperative radiotherapy of the tumor bed with additional radiotherapy of the regional lymph nodes (6/17 after LAD); while in the stage I-II MCC, only 16 patients (40%) received radiotherapeutic treatment of the regional lymph nodes (two patients had previous LAD; one patient had LAD without LNI). Accordingly, comparison of the two different treatment groups (postoperative radiotherapy of the tumor bed with or without regional lymph node irradiation) revealed highly significant differences of baseline characteristics in regard to the prognostic factors AJCC-stage and SLNB result (*p* < 0.001), with worse prognostic factors in the group which received additional LNI. Other baseline characteristics that could influence prognosis, especially age, sex, previously performed LAD and resection status were similar in both cohorts. Surprisingly, however, the better prognostic factors concerning AJCC-stage and SLNB result in the group not receiving LNI, and do not translate into better outcomes in the present study. PFS and OS are similar for both cohorts, with 1- and 3-year OS rates of 93.9% and 68.8% in the cohort with LNI and 91.7% and 68.6% in the cohort without LNI (*p* = 0.9) and 1- and 3- year PFS rates of 66.1% and 59.2% versus 64.3% and 54.4% in the group with and without LNI, respectively (*p* = 0.8). In our opinion, this counterintuitive result is most likely attributed to the more intensive treatment regimen with additional radiotherapy of the regional nodes, leading to a relatively improved outcome of the MCC stage III patients. Of course, methodological impairments like small sample size and the frequently imprecise pathological reports, which resulted in a number of patients, who could retrospectively only be classified as AJCC stage ≤ II could lead to a potential bias.

In univariate and multivariate analyses, however, significant factors associated with survival outcome were age (PFS) and age and sex (OS). However, AJCC stage and if a LNI was done was not associated with PFS or OS. For regional recurrence-free survival there was a trend for a benefit from LNI. This fits to observations from other cancer types such as melanoma that regional surgery or radiation can help to control local recurrence but does not influence survival of patients. This demonstrates the need of systemic adjuvant therapy e.g., with anti-PD1 antibodies. Recently, it was shown that adjuvant nivolumab given every 4 weeks for a year in patients with completely resected stage I-IV MCC improves disease free survival by 50% compared to observation (HR 0.53; 95% CI 0.26–1.06; *p* = 0.07) [24].

Most of the only clinically staged patients received elective irradiation of the lymph node basin. Only three patients without surgical (twice stage IB, once stage IIB) staging did not receive elective lymph node irradiation because of age and comorbidities. However, two of them (both stage IB) experienced early regional relapse, after 3 and 15 months. These are very small patient numbers, but it supports the recommendation of elective irradiation of the lymph node basin for clinically staged I-II MCC.

In the one randomized controlled trial on elective irradiation of the regional lymph node basin in MCC, Jouary et al. reported low skin toxicity from radiation therapy, which was mainly observed on the primary tumor’s bed area with 19.3% grade I and 7.2% grade II skin toxicity of the whole cohort. No significant difference was found between the group with or without lymph node irradiation (*p* = 0.051). In this context, and since most of the patients have to undergo adjuvant treatment of the tumor bed anyway, a generous recommendation for elective lymph node irradiation seems reasonable.

### 4.2. Patterns of Relapse

Analysis of the pattern of relapse show that the most frequent sites of relapse are the regional nodes with 69.2% involvement of regional nodes of all observed tumor recurrences (9/13 patients) and that regional relapse is more frequent in the cohort without LNI (OR 2.28, *p* = 0.11), however, these findings do not reach significance due to small patient numbers. Nevertheless, it is an interesting fact that patients treated with LNI showed significantly more distant recurrence but less regional recurrence. Considering the higher rate of distant recurrence as indicator for the higher aggressiveness of MCC in the cohort with LNI, due to higher AJCC stages, this could indicate that LNI was effective to prevent a similarly higher expected rate of regional recurrence in this cohort.

### 4.3. Sentinel Node-Negative Patients

Comparison of postoperative radiotherapy with or without additional RT of the regional lymph nodes for patients with negative SLNB only showed even slightly better OS and PFS for the patients that received additional radiotherapy of the lymph nodes with 1- and 5-year OS of 100% and 80.0% versus 95.0% and 73.7%, and 1- and 3-year PFS of 88.9% and 77.8% versus 67.2% and 61.1% for the cohort with or without LNI, respectively, however, this was statistically nonsignificant (Figure 6). Tumor recurrence occurred equally often in both cohorts, but relative risk for relapse being located in the regional nodes was 2.4 for the cohort without LNI. These findings indicate that on the one hand, relapse in the regional nodes is the most frequent site of relapse even if the sentinel node was negative, and on the other hand, that radiotherapy of the regional lymph nodes might be associated with a lower risk of regional relapse, although nonsignificant due to small patient numbers. This could be due to an underestimated rate of false-negative SLNB results, since, to our knowledge, the accuracy of SLNB in MCC was not yet investigated systematically and proven with certainty [1]. All in all, postoperative radiotherapy seems to be highly effective since infield-recurrence was only observed in 5.3% of all patients. However, defining the target volume of regional nodes can be challenging, especially for tumors located in the facial area, which can result in outfield-recurrence in regional lymph nodes or in-transit metastasis despite previously performed lymph node irradiation.

### 4.4. Radiation Monotherapy for Microscopical Lymph Node Involvement

German as well as NCCN treatment guidelines for MCC recommend LAD and/or radiation to the nodal basin for patients with positive SLNB. In the present study, 7 patients underwent radiation monotherapy for microscopical lymph node involvement. Aside from one patient that experienced outfield-locoregional recurrence under radiotherapeutic treatment and died due to sudden cardiac death within 3 months, all of the other patients (6/7 patients) stayed recurrence-free until after a median follow-up of 42 months. This indicates that radiation monotherapy is a treatment option instead of LAD for patients with positive SLNB and is in line with the findings of Fang et al., who report an excellent regional control rate of 100% after radiation monotherapy of microscopically involved lymph nodes after a median follow-up of 18 months [25].

### 4.5. Limitations

Since this is a retrospective analysis of a rare malignant skin tumor, it comes with several limitations, especially that the small size of the cohort causes a primarily descriptive analysis and that significance is frequently not reached. Furthermore, to be mentioned is the heterogeneity in regard to clinical or surgical staging, primary tumor localization, different radiotherapeutic treatment approaches with brachytherapy or EBRT and, as previously mentioned, the frequently imprecise pathological reports, which resulted in a number of patients, who could retrospectively only be classified as AJCC stage ≤ II. Another relevant methodological impairment due to the retrospective design is the missing valid data regarding immune competence of the patients and the association with Merkel cell polyomavirus (MCPyV). Since immunodepression and association with MCPyV are prognostic factors in MCC, this could lead to an unnoticed bias. However current data on elective lymph node irradiation in MCC with respect to SLNB is sparse due to rareness of the disease and therefore the present study can contribute to improvements in therapy despite the mentioned limitations.

## 5. Conclusions

Our data show that regional recurrence is the most frequent site of relapse in stage I-III MCC treated with curative-intended primary resection followed by postoperative radiotherapy and that elective irradiation of the regional lymph nodes reduces the risk of regional relapse even if the sentinel lymph node was negative. In the context of relatively low toxicity of elective lymph node irradiation [16], we conclude that a more generous recommendation for elective lymph node irradiation seems reasonable, even if the sentinel lymph node was negative. Furthermore, our data support previous studies that suggest radiation monotherapy as a treatment option for patients with microscopical lymph node involvement. Larger data sets will be necessary to validate these conclusions and to determine whether a reduction of regional relapse would translate into improved PFS and OS.

## Figures and Tables

**Table 1:** Patient characteristics of 57 patients with postoperative radiotherapy for Merkel cell carcinoma (MCC), divided into total cohort and subgroups receiving either radiotherapy of the tumor bed without lymph node irradiation (without LNI) or with additional irradiation of the lymph nodes (with LNI).

		Total Cohort (n = 57)	with LNI (n = 33)	without LNI (n = 24)	*p*-Value
median age (range) in years		73 (50–89)	73 (50–88)	72.5 (52–89)	0.517
sex	male	33 (57.9%)	20 (60.6%)	13 (54.2%)	0.629
	female	24 (42.1%)	13 (39.4%)	11 (45.8%)	
AJCC-stage	I	13 (22.8%)	5 (15.2%)	8 (33.3%)	<0.001
	≤II	19 (33.3%)	8 (24.2%)	11 (45.8%)	
	II	8 (14.0%)	3 (9.1%)	5 (20.8%)	
	III	17 (29.8%)	17 (51.5%)	0 (0%)	
SLNB	done	42 (73.7%)	22 (66.7%)	20 (83.3%)	0.162
	not done	15 (26.3%)	11 (33.3%)	4 (16.7%)	
SLNB result	positive	12 (21.1%)	12 (54.6%)	0 (0%)	<0.001
	negative	30 (52.6%)	10 (45.5%)	20 (100%)	
lymph node dissection	done	9 (15.8%)	8 (24.2%)	1 (4.2%)	0.064
	not done	47 (82.4%)	24 (72.7%)	23 (95.8%)	
	unknown	1 (1.8%)	1 (3.0%)	0 (0%)	
resection status (R-status)	0	52 (91.2%)	29 (87.9%)	23 (95.8%)	0.073
	1	4 (7.0%)	4 (12.1%)	0 (0%)	
	X	1 (1.8%)	0 (0%)	1 (4.2%)	
primary tumor localization	craniofacial	19 (33.3%)	14 (42.4%)	5 (20.9%)	0.185
	body trunk	6 (10.5%)	4 (12.1%)	2 (8.3%)	
	extremities	32 (56.2%)	15 (45.5%)	17 (70.8%)	

**Table 2:** Sites of relapse for the total cohort and the cohorts with or without irradiation of regional lymph nodes (LNI) in absolute and relative frequencies. One patient experienced local and regional failure simultaneously (in the cohort without LNI), two patients experienced regional and distant failure simultaneously (both in the LNI cohort).

Site of Relapse	with LNI (n = 33)	without LNI (n = 24)	Total (n = 57)
Local	1 (3.0%)	1 (4.2%)	2 (3.5%)
Regional	3 (9.1%)	6 (25.0%)	9 (15.8%)
Distant	5 (15.2%)	0 (0%)	5 (8.8%)

**Table 3:** Sites of relapse of the patients with negative SLN in absolute and relative frequencies, divided into subgroups that received additional lymph node irradiation (with LNI) or irradiation of the tumor bed only (without LNI). One patient had local and regional failure simultaneously (in the cohort without LNI), one patient had regional and distant failure simultaneously (in the LNI cohort).

Site of Relapse	Patients with Negative SLNB Only		
	with LNI (n = 10)	without LNI (n = 20)	Total (n = 30)
Local	0 (0%)	1 (5%)	1 (3.3%)
Regional	1 (10%)	4 (20%)	5 (16.7%)
Distant	2 (20%)	0 (0%)	2 (6.7%)

## Data Availability

The authors confirm that the data supporting the findings of this study are available within the article. Due to the nature of this research, participants of this study did not agree for their data to be shared publicly, so supporting data is not available.
